# Post-Operative Headache and Psychomotor Agitation in the Recovery Room Caused by Subarachnoid Hemorrhage after Orbital Decompression Surgery

**DOI:** 10.5812/kowsar.22287523.1473

**Published:** 2011-07-01

**Authors:** Babak Kavand, Mohammad Reza Ghodrati, Saeid Safari

**Affiliations:** 1 Department of Anesthesiology, Tehran University of Medical Sciences, Tehran, IR Iran

**Keywords:** Subarachnoid Hemorrhage, Postoperative Period, Psychomotor Agitation, Headache, Surgical Decompression

## Abstract

There are several etiologies for agitation and headache in the recovery room, including preoperative or intraoperative drug administrations, physical disorders such as painful surgical procedures, and finally, unusual consequences of some operations such as subarachnoid hemorrhage after orbital decompression, a disastrous complication that should be particularly considered whenever facing any sort of headache or unattainable full recovery. In this study a rare case of subarachnoid hemorrhage is presented.

## 1. Introduction

**Figure 1.**
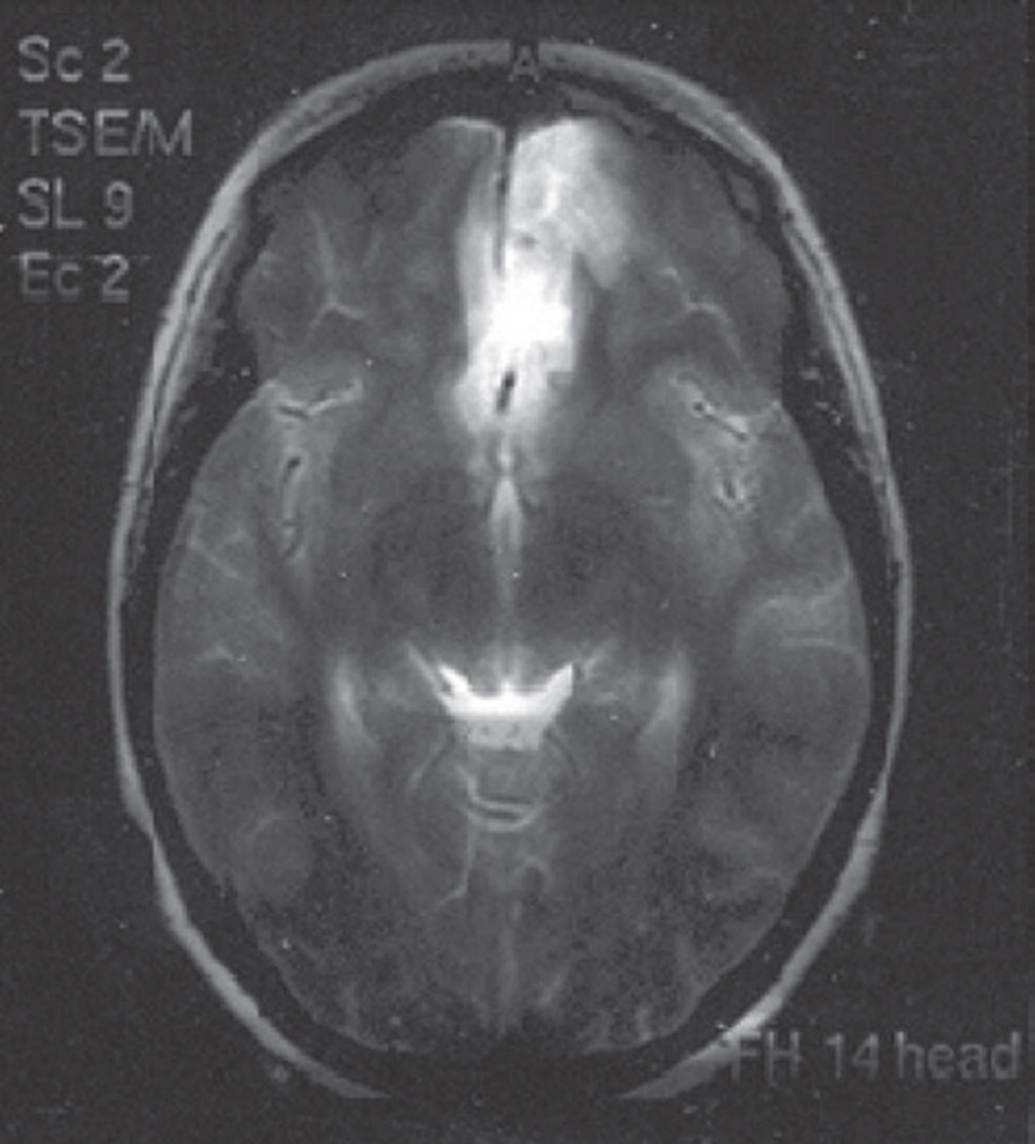
Axial T1, T2, PD, MRI. Diffuse Subarachnoid Hemorrhage; Ischemic Changes in the ACA Territory

**Figure 2.**
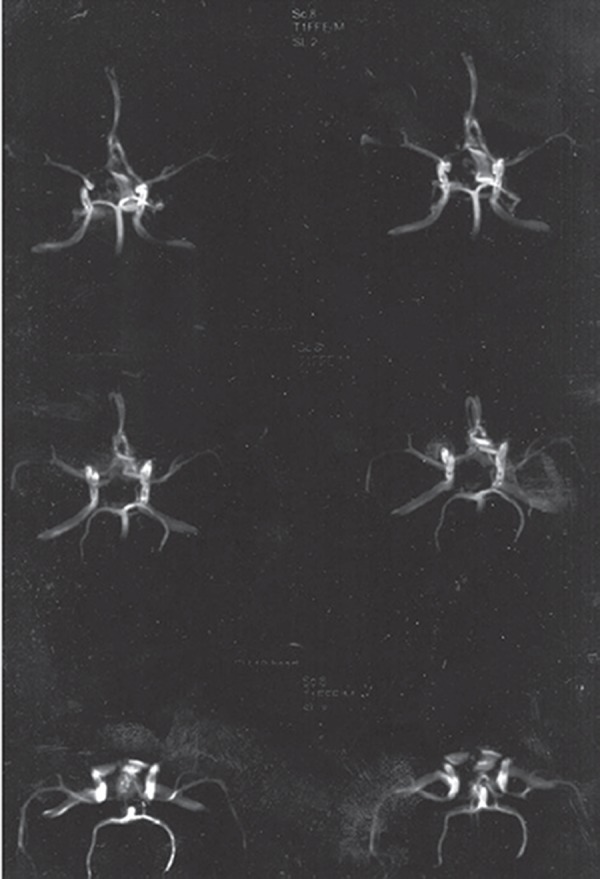
MRA with TOF Technique. No Evidence of Aneurisms or AVM

Patients recovering from a variety of surgical procedures sometimes exhibit inappropriate mental reactions, ranging from lethargy and confusion to physical combativeness and extreme combativeness that, aside from disturbances to staff and other patients, have significant medical consequences for the patient. Among the most common causes of postoperative agitation are narcotics, alcohol and cocaine abuse, certain drug-related side effects (such as scopolamine and etomidate), postoperative pain, urinary retention, gastric expansion, urinary and gastric catheters, an endotracheal tube, and respiratory disorders (e.g., hypoxia and respiratory acidosis). Other causes are neurologic, sometimes resulting in seizures in patients with epilepsy and head trauma. In this study, we present a rare case of severe disorientation and headache after orbital decompression surgery due to subarachnoid hemorrhage in a patient with Grave’s disease. The patient was a 25-year-old woman who had been diagnosed with Grave’s disease in early 2010; on the occasion of this case study she presented with bilateral exophthalmia and ophthalmopathy in progress. As a consequence of radioactive iodide treatment, she had been prescribed a Levothyroxine tablet of 50 μg daily for treatment of hypothyroidism. Her thyroid function tests from 01/11/2009 (Total T4 = 32.5 nmol/L (45-120), TSH > 40 (0.32-5.2), and T3 = 0.85 nmol/L (0.5-1.5) represented hypothyroidism, so orbital decompression surgery was postponed until euthyroidism was reached. On 03/07/2010, thyroid function was evaluated again, and all levels were reported to be normal. Other preoperative routine tests were normal, as well. In the patient’s physical examination, nothing significant except bilateral exophthalmia was detected, and thyroid size was normal. Vital signs were in the normal range, and no important history of individual or family medical illness was reported. There was no history of opium or alcohol abuse and no smoking habit. On 04/07/2011, the left side of the orbital decompression operation was carried out and lasted two hours. Hydrocortisone (100 mg), fentanyl (100 μg), and midazolam (1 mg) were administered as premedication, and thiopental (350 mg) and atracurium (30 mg) were administered for anesthesia induction. Intubation was performed with a cuffed PVC orotracheal tube (tube 7 mm) and anesthesia maintained with propofol (100 μg /kg /min) plus atracurium and fentanyl in appropriate intervals and dosages. Lactated ringer (1500 mL) was infused through the operation and standard monitoring including ECG, NIBP, capnography, and pulse oxymetry were applied. No hemodynamic or respiratory complications were reported during surgery and no signs of light anesthesia or spontaneous breathing were detected. Controlled mechanical ventilation with 600 mL tidal volume and RR = 12 was restored. According to the ophthalmologic plastic surgeon’s note, an ethmoidectomy was conducted and the medial half of the maxillary sinus was removed. At the end of the operation, anesthetic drugs were stopped and muscle relaxation was reversed with neostigmine (2.5 mg) and atropine (1.25 mg). The trachea was extubated and with acceptable consciousness the patient was transferred to the recovery room. In the recovery room, the patient gradually developed severe agitation and disorientation, while the general and neurological physical examinations and the vital signs were normal. Upon regaining modest awareness, she complained of severe frontal and vertex headache. To palliate the symptoms the following medications were injected intermittently (along with oxygen 5 L/min with mask): haloperidol (5 mg), meperidine (30 mg), fentanyl (50 μg), midazolam (2 mg), and morphine (3 mg). She remained under observation in the recovery for four hours, and no any neurologic signs demonstrating sensory or motor abnormality were detected. Her symptoms improved a little but a moderate headache persisted when transferred to the ophthalmology ward, where she gradually lost the ability to move her right lower limbs and developed considerable stiffness in the neck. In the ophthalmologic examination, symmetrical papillary size and reaction with no retinal bleeding were noted. On 06/07/2010, a psychiatric consultation was requested for severe restlessness and right lower limb paresis suspecting conversion disorder. Psychosomatic problems were ruled out, and according to a later neurologic consultation, the patient was reported to be conscious but agitated, was able to obey orders, was experiencing neck rigidity, and had a right lower limb force of one fifth and a deep tendon reflex of 2 to 3 plus. Additionally, in the requested CT scanning, a hypodensity in the territory of the anterior cerebral artery and subarachnoid hemorrhage in the left frontal area was obvious, and an ICU admission ordered. On the same day, a neurosurgical consultation reported Achilles’ hyperreflexia, and extensor right foot reflex and subarachnoid hemorrhage was diagnosed. Eventually on 08/07/2010, the patient was sent to the ICU, and medical treatment was undertaken. In the ICU she suffered from severe headache episodes, photophobia, delirium, and right lower limb weakness. Thorough, frequent physical examinations were normal, and abnormal sensory findings were not reported. In fundoscopic examinations, pupil edema and subhyaloid hemorrhage were not detected. A brain magnetic resonance imaging (MRI) with and without contrast revealed subarachnoid hemorrhage, and some clues of ischemic changes in the territory of the anterior cerebral artery were evident (Figure 1, 2).

All echocardiographic cardiac indexes and valvular conditions were reported to be normal. Two weeks later, a delayed brain digital subtraction angiography (DSA) of four vessels showed no aneurismal pathology, and a subsequent brain CT angiography was normal as well. During the patient’s stay in ICU, the headache gradually subsided, and at the time of discharge on 26/07/2010, the weakness in the right lower limbs had improved. On the patient’s second visit to the neurosurgical clinic (08/08/2010), no neurologic findings were observed, lower limb force had improved completely, and the results from a repeat brain CT angiography were normal.

## 2. Discussion

For a short period after regaining consciousness after anesthesia some patients appear unable to appropriately process sensory input. Most patients exhibit somnolence, slight disorientation, and sluggish mental reactions that pass rapidly. Others experience erratic emotional swings such as weeping and an escalated resistance to positioning and restraint. Patients with mental retardation, psychiatric disorders, organic brain dysfunctions, or hostile preoperative interactions are particularly likely to manifest these problems after surgery. Ethnic, cultural, and psychological characteristics play some roles as well (1). Recall of intraoperative events can generate severe pain and anxiety (2). Disorientation, paranoia, and combativeness may occur after the use of scopolamine as a premedication or antiemetic. It is important to check for unusual pain sources such as corneal abrasions, entrapment of body parts, or small devices left beneath the patient. Other causes of aggressiveness and agitation include nausea, dizziness, pruritus, moderate hypoxemia, respiratory acidosis, limitation of respiratory volume (by chest dressings and gastric distention), and inability to produce a forceful cough or clear secretions. Lactic acidosis, hypoxemia, and cerebral hypoperfusion are among the other causes of agitation. Seizure activities might mimic similar behaviors (1). Altered mental status is treated supportively because most emerging reactions disappear within 10 to 15 minutes, allowing patients to choose their own position. It is also important to identify whether a patient is reacting to pain or anxiety. Benzodiazepines and barbiturates, for instance, are ineffective analgesics, and opioids are poor sedatives (1). Concerning severe postanesthesia headache, the reported incidence ranges from 12 to 35%, but up to 60% of patients complain of some level of headache, and most investigations have failed to identify a single agent as being responsible for headaches after general anesthesia. Individuals who are susceptible to headaches caused by stress or other factors are more likely to complain of postoperative headaches (3). Concerning the patient’s symptoms in our case study, although all of the above causes cannot be excluded, successive clinical findings and of course also laboratory data revealed subarachnoid hemorrhage and resulting vasospasm; these are rare complications of orbital decompression but it seems highly probable that they explain her recovery scenario. The documented complications of orbital decompression currently include diplopia, strabismus, infraorbital anesthesia, epiphora, obstructive sinus disease, dacryostenosis, medial entropion, visual loss, meningitis and CSF leaks (4-10). To our knowledge, not more than two cases of subarachnoid hemorrhage have been reported as a complication of decompression surgery (11). In these reported cases, dural penetration resulted in subarachnoid hemorrhage-induced vasospasm and ischemia. Spontaneous (nontraumatic) subarachnoid hemorrhage due to ruptured aneurisms or an arteriovenous malformation has been described very well from a clinical perspective and traumatic types. Other explanations of blood in subarachnoid space include intracranial hemorrhages, embolic cerebral attacks and head traumas. Clinically, subarachnoid hemorrhage usually causes severe and generalized headache, and a lack of headache precludes the condition’s diagnosis. Loss of consciousness is frequent, as are vomiting and neck stiffness. Blood pressure and temperature frequently rise as a result of the hemorrhage. Nuchal rigidity and other evidence of meningeal irritation are common. Focal neurologic signs such as hemiparesis occur occasionally (12). With respect to laboratory findings, a CT scan will usually confirm the diagnosis, and the CT scan is most sensitive in patients with altered consciousness. Aneurysms may not be evident on the CT scan. MRI is especially useful in detecting small AVMs localized to the brain stem. If the CT scan fails to confirm the diagnosis of subarachnoid hemorrhage, lumbar punctures and CSF examinations are performed. Once the diagnosis is documented, “four vessels” cerebral angiography of both carotids and vertebral arteries is undertaken to visualize the entire cerebral vascular anatomy. Because multiple aneurysms occur in 20% of patients and AVMs are frequently supplied from multiple vessels. Hypertensive intracerebral hemorrhages are also manifested by obtundation and hemorrhagic spinal fluid. Bacterial meningitis and ruptured mycotic aneurisms are also among differential diagnoses. Of the subarachnoid hemorrhage complications that highly explain our case, the clinical findings are arterial vasospasm, delayed arterial narrowing occurred in vessels surrounded by subarachnoid blood and can lead to parenchymal ischemia. Clinical ischemia typically does not appear before the fourth day after the hemorrhage and then spontaneously resolves. The diagnosis can be confirmed by cerebral angiography. The severity of the spasm is related to the amount of blood in subarachnoid space and therefore is lesser after AVM and trauma. Other subarachnoid hemorrhage complications are hydrocephaly and seizures (12). Medical treatment consists of preventing intracranial pressure and blood pressure increments. The patients should be under intensive care and ordered to complete bed rest with the head of the bed elevated 15–20 degrees. In hypertensive cases, it is prudent to reduce blood pressure to approximately 160/100 mmHg on admission. Hypotension should be prevented, however. Intravenous fluids should be administered with care because overhydration can exacerbate cerebral swelling. To reduce the consequences of vasospasm, oral nimodipine is advised. Vasospasm is treated by induced hypertension with phenylephrine or dopamine.

## 3. Conclusion

Subarachnoid hemorrhage, cerebral vasospasm, and ischemia or infarction can be grave and even fatal after orbital decompression surgery. Not more than two cases have been explored in the literature as of the time of this writing. It is important for anesthesiologists to be aware of any alterations in consciousness and focal neurologic signs after operations in which dural rupture is highly probable.
